# Supplementary material for: Single-Cell Transcriptomics Reveals the Complexity of the Tumor Microenvironment of Treatment-Naive Osteosarcoma
Source: Front Oncol. 2021 Jul 21;11:709210. doi: 10.3389/fonc.2021.709210 (PMC8335545; doi:10.3389/fonc.2021.709210)
Supplement: Supplementary Document 1 — Kaplan-Meier curve of the 518 genes associated with survival. [file DataSheet_1.zip › Supplementary Document 1/sur.RAB38.pdf]

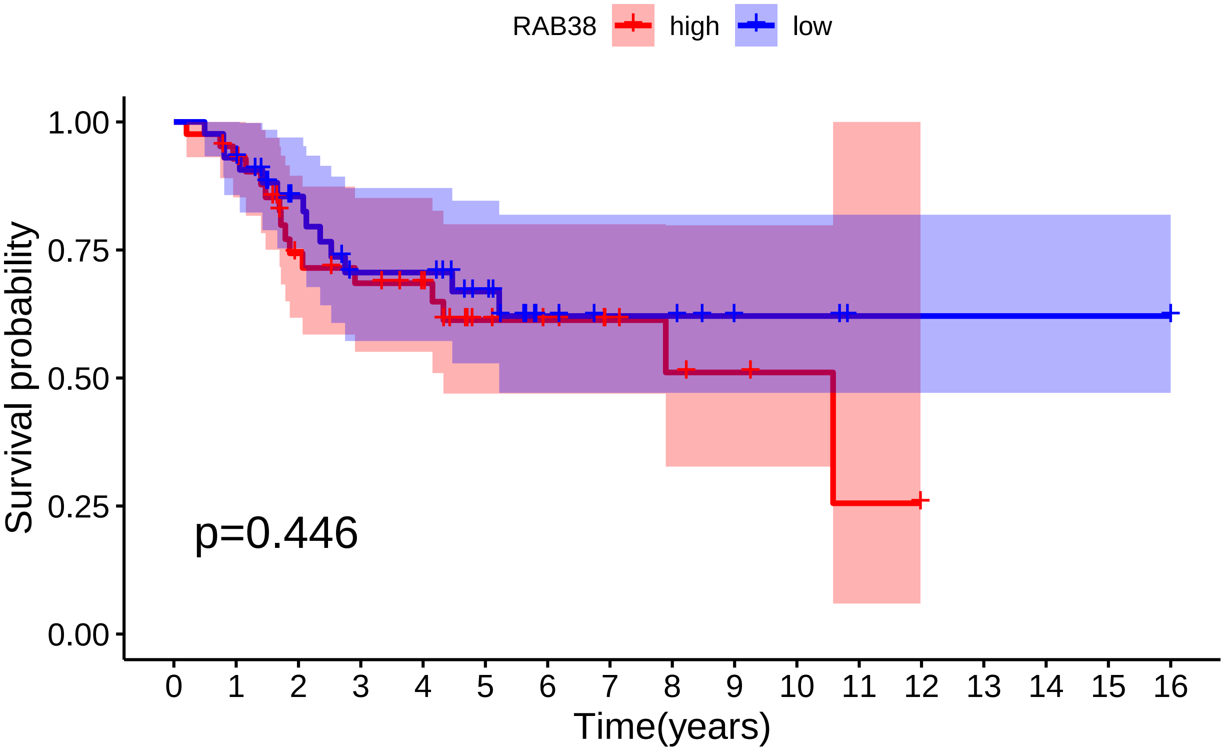

RAB38	0	1	2	3	4	5	6	7	8	9	10	11	12	13	14	15	16
high	42	38	26	23	20	12	10	7	5	4	2	1	0	0	0	0	0
low	43	40	29	22	22	16	8	6	6	3	3	1	1	1	1	1	1

Time(years)
